# Nanotoxicology and Metalloestrogens: Possible Involvement in Breast Cancer

**DOI:** 10.3390/toxics3040390

**Published:** 2015-10-28

**Authors:** David R. Wallace

**Affiliations:** Oklahoma State University Center for Health Sciences, Department of Pharmacology & Physiology, Tulsa, OK 74107-1898, USA; E-Mail: david.wallace@okstate.edu; Tel.: +1-918-561-1407; Fax: +1-918-561-5729

**Keywords:** cadmium, metalloestrogen, heavy metal, aluminum, titanium, silicon, silver, nanotoxicology

## Abstract

As the use of nanotechnology has expanded, an increased number of metallic oxides have been manufactured, yet toxicology testing has lagged significantly. Metals used in nano-products include titanium, silicon, aluminum, silver, zinc, cadmium, cobalt, antimony, gold, *etc.* Even the noble metals, platinum and cerium, have been used as a treatment for cancer, but the toxicity of these metals is still unknown. Significant advances have been made in our understanding and treatment of breast cancer, yet millions of women will experience invasive breast cancer in their lifetime. The pathogenesis of breast cancer can involve multiple factors; (1) genetic; (2) environmental; and (3) lifestyle-related factors. This review focuses on exposure to highly toxic metals, (“metalloestrogens” or “endocrine disruptors”) that are used as the metallic foundation for nanoparticle production and are found in a variety of consumer products such as cosmetics, household items, and processed foods, *etc.* The linkage between well-understood metalloestrogens such as cadmium, the use of these metals in the production of nanoparticles, and the relationship between their potential estrogenic effects and the development of breast cancer will be explored. This will underscore the need for additional testing of materials used in nano-products. Clearly, a significant amount of work needs to be done to further our understanding of these metals and their potential role in the pathogenesis of breast cancer.

## 1. Introduction

The term “nanomaterial” or “nanoparticle” is defined by its size and implies a very small structure. Originally, the name and size were derived from the particle size of the material, being 10^−9^ meters or one nanometer in size [1,2]. Currently, the nanomaterial size range is usually from 1–100 nm. Sizes up to 1000 nm have been reported, so the actual size is a relative term [3]. A list of basic terms based on particle function include; (1) nanofoil, (2) nanopowders, (3) nanoprisms or crystals, (4) nanospheres, (5) quantum dots, and (6) nanowires or rods, to name a few. Classes of nanomaterials are divided into (1) natural and (2) synthetic materials. Synthetic materials can then be further subcategorized into organic (carbon-based) and inorganic (non-carbon based, such as silicon, titanium, *etc.*). For this review, the emphasis will be on the synthetic inorganic/non-carbon-based nanomaterials, such as heavy metals. The potential use of inorganic nanomaterials varies significantly across a wide range of consumer products [1,4,5]. There has been a significant rise in the usage of nanomaterials in food products, cosmetics, and drugs. One prototypical “nanomaterial” found in a “cosmetic” product is zinc oxide, which is found in sunscreen, usually combined with a form of titanium [4]. Transdermal absorption is minimal, and it is accepted that zinc/titanium nanoparticles pose a minimal health risk in this form.

Nanotechnology has provided us with an increasing number of nanoparticle-based consumer products (Table 1). There are numerous products that utilize nanotechnology and nanoparticles that are available for over-the-counter purchase. In most instances, the consumer is not even aware of the existence of nanoparticles in the product they are using. The use of nanoparticles and nanomaterials in food represents a growing sector of the nanotechnology market. Sensor technology for the identification of bacteria/toxins or other impurities has been utilized. Although the uses of nanotechnology in food products could be extensive, modifications are still controlled by the Food and Drug Administration. Estimates suggest four new food-related compounds enter the marketplace each week [5]. Since 2005, there have been nearly 1700 compounds added to the Project on Emerging Nanotechnologies (PEN), which represents a 24% increase compared to 2010 [5]. As the explosion of additional uses for nanoparticles continues, consideration for the ethical and moral concerns has lagged behind [6]. Consideration for the social, economic, and environmental impact of nanoparticles needs to be considered as products are developed, not *post hoc*.

In general, the safety of nanoparticles is not well understood for most compounds, and the environmental impact of the manufacturing and disposal of nanoparticles has not been thoroughly examined [1,7,8]. Proper safety testing and analysis of these compounds has been incomplete at best [9]. In many instances, the pathophysiological activity of these particles can be entirely different from their larger molecular counterparts. The smaller particle size may lead to increased cellular absorption and/or transport [10]. The rate of transport may also increase due to the smaller molecular size. Once inside the cell, the precise effects of these compounds are only now beginning to be understood. DNA damage and oxidative stress are common cellular pathways for the development of pathologies following exposure to various nanoparticles and metal oxides [11,12,13]. Each of these metals will be thoroughly examined in their individual sections.

**Table 1:** Examples of commercial nanomaterial use, and the products impacted by nanomaterials.

Uses	Material
**Surface/coating**	Ceramics and scratch-resistant surfaces.
**Textiles**	Wrinkle-free and water-resistant.
**Sports**	Protective gear/helmets and antimicrobial material in fitness areas (silver and copper oxides, carbon nanotubes).
**Foods/Edibles**	Preservatives (mainly titanium dioxide).
**Cosmetics**	Sunscreens (zinc oxide).
**Military**	Biosensors—detect hazardous materials.
	Uniform material—lightweight, thermal protection and camouflage, projectile resistance.
	Communications—lightweight and can be easily worn in normal garments.
	Medical—sensors in wearable materials.
	Weapons—lightweight, increased destructive force.
**Aerospace**	Lighter and more durable material for aircraft.
**Chemistry/synthesis**	Catalyst for chemical reactions.
**Construction**	Wood—improved sustainability and endurance of wood-related products.
	Steel—improved tensile strength, durability, and longevity.
	Concrete—improved strength and composition particle size for alumina and silica.
	Glass—use of titanium and silica oxides to improve cleaning ability and fire protection.
	Coatings.

Ref. [1,5,14,15] as well as references and publications within.

The class of compounds referred to as endocrine disruptors can be broken down into subclasses based on their structure and/or function [16]. Structurally similar compounds can be phenolic or non-phenolic, referencing the “A” ring on endogenous estrogen. Chemicals displaying estrogenic functionality that are not chemically similar to estrogen may act directly or indirectly to affect estrogen receptor signaling. Compounds in the “non-chemically” similar class include food additives, pesticides, plasticizers, and environmental pollutants, including some heavy metals [16]. When examining multiple signaling pathways associated with estrogen signaling, there is a significant level of complexity that involves the interrelationship between many genes [16,17]. Changing the chemical structure or composition of metal-containing particles (valence state, organic or inorganic form, *etc.*) will alter their biological availability and toxicity. Transitional metals may catalyze oxidative/reductive reactions, such as the Fenton and Haber-Weiss reaction, leading to the generation of reactive oxygen species [18]. Elevated levels of hydroxyl radicals, in the presence of transitional metals, lead to lipid bilayer damage, fragmentation of DNA, and oxidation of cellular proteins [18]. Metals used in industry settings can end up in the food supply, groundwater, drinking water, and soil. There are also forms of different metals found in commercial preparations. These metals include copper, cobalt, nickel, lead, mercury, tin, chromium, cadmium, aluminum, vanadate (metal anion), antimony, barium, selenite, and arsenite. Cadmium is in many farm fertilizers and can make its way into soil and water. Some of the other main sources of cadmium include cigarette smoke, rechargeable batteries, certain cosmetics, bread and cereals, potatoes, root crops, and vegetables. Cadmium is a widely distributed metal used in manufacturing and present in some consumer products. It is used as a metal alloy, a paint additive, and in batteries (Ni-Cd), pigments, metal coatings, plastics, welding, and battery manufacture. Numerous heavy metals are implicated in the development of a variety of cancers (arsenic, beryllium, cadmium, nickel, and hexavalent chromium, plus others). There has been an increasing number of studies that have shown that cadmium may facilitate breast cancer development [19,20,21,22,23,24,25,26,27]. Evidence exists that the route of exposure and the chemical state of cadmium are important to tumorigenesis following exposure [28]. Nearly two decades ago, the presence of heavy metals as a potentially toxic addition to inorganic pesticide mixtures was addressed [29]. They reported that multiple metals such as cadmium, cobalt, copper, and zinc are found at relatively high levels as impurities in pesticide preparations. In other preparations, higher levels of lead, nickel, iron, and manganese were observed [29].

The intent of this paper is not to provide an exhaustive review of the literature that would be significant. Instead, the primary aim and purpose is to provide a brief overview of the use of nanoparticles alone, or collectively as nanomaterials, and the current state of the literature as it pertains to the possible relationship between nanoparticles/metals and the development of breast cancer. Collectively, metals used in a variety of nanotechnology have been reported to promote the formation of breast cancer, whereas other metals have been utilized as an anti-cancer treatment. The relatively new field of nanotoxicology (the toxicity of nanoparticles/nanomaterials on biological systems) is rapidly growing due to the sparse amount of toxicity data that is currently available regarding these compounds.

## 2. Metals and Nanomaterials

### 2.1. Treatment of Cancer

Although the primary focus of this review is the potential toxicity of metal oxides that make up nanoparticles [30,31,32], an increasing body of evidence suggests that “noble” metals may provide effective anticancer therapy. Metals that have been used (platinum, palladium, ruthenium, gold, silver, copper, rhodium, iridium, osmium, and rhenium) for the treatment of cancer have demonstrated various degrees of effectiveness [33]. The use of metals in medicine is centuries old. Gold, arsenic, and silver are some of the key metals reported in the ancient literature (Greek, Roman, and Chinese) as having valuable medicinal properties. As anticancer drugs, the chemical derivation of platinum (cisplatin) was shown to have potent anticancer activity, treating both testicular and ovarian cancer [34,35]. Very recently, a report from the University of Warwick described the action of osmium and concluded that osmium is nearly 49 times more potent than cisplatin in destroying tumor cells [36]. Medici *et al.* [33] note that numerous preparations are available on the Internet, containing a variety of heavy metals in differing chemical states as potential treatments for an array of diseases. Concerning cancer treatment, nanoparticles have exhibited multiple uses [37]. Utilizing the potential toxicity of the particles, nanoparticle preparations are used for improved imaging of tumors due to their increased uptake and concentration in the cancerous cells [37,38]. Chemical structure specialization of the nanoparticle permits the binding and transport of anticancer drugs directly to the tumor. The use of gold particles has been particularly useful in this regard, as well as in improving the detection of tumorous cells [1,5,14,15,38]. It is becoming clear that there are therapeutic uses for some heavy metals (such as gold in the detection of tumor cells), yet caution is needed when considering the use of other transitional metals.

### 2.2. Lack of Toxicity Testing and Potential for Pro-Cancer Properties

The conventional way of thinking has come under scrutiny when trying to estimate toxicity associated with nanoparticles [39,40]. Nanoparticle toxicity tends to follow the relationship of size-dependent toxicity instead of the usual dose-/concentration-dependent toxicity. Examining the toxicity of bulk metal silver, for example the total exposure to the metal, the daily exposure, the route of exposure, and from that information and the examination of animal and/or human models we can determine toxicity associated with that silver exposure. A recent renovation of that concept has concluded that the conventional means of determining toxicity are not adequate concerning dose/concentration, but instead the surface area of the nanoparticle would be the factor to be considered [39,40]. Rethinking the methods for determining risk assessment should include the following: (a) aerosol standards and key nanoparticle metrics; (b) dependable exposure scenarios and affordable monitoring technologies, exposure data, and modeling systems; and (c) biomedical data on nanoparticle translocation and toxicity [2,39]. These changes would have a significant impact on the development of drugs based on nanoparticles since they do not follow conventional toxicity testing. Changes in the manner we examine the safety-, risk- and cost-effectiveness of nanoparticle-based drugs are necessary, yet may increase the overall cost of these drugs [41].

Very effect methods of analyzing nanoparticle toxicity include computational modeling [42] and quantitative-structure-activity-relationships (QSAR) [43,44]. An advantage to using QSAR is animal models may be by-passed, which avoids the need to correlate findings in animal models to predict human responses. The use of animals may require lengthy experimentation and validation as an appropriate human model [44]. Also, the use of QSAR allows for quickly filling voids in our understanding of nanoparticle toxicity [43] as well as ameliorating the risks/hazards for a particular nanoparticle. Using the derived QSAR data, a better grouping of nanoparticles can be completed [45]. An outcome-based grouping of nanoparticles instead of chemical structure will improve our understanding of their adverse effects and facilitate exposure limitations [45]. By using available information on the chemico-physiological properties of different metals, computational modeling (or “*in silico*” experimentation) can be performed. Currently, there have been *in silico* studies performed on titanium, copper, zinc, and iron (in their respective oxide form) which have aided our toxicological understanding of these nanoparticles [42].

An excellent review article has been published by Buzea *et al.* [3] that elegantly explains the cycle of nanoparticles from synthesis to transport to exposure to toxicity. Their report starts with various types of manufacturing known to generate nanoparticles, and continues on to the use of nanoparticles in consumer products [3]. What the Buzea report and other reviews have stressed is that improved guidelines/regulations are needed for the proper manufacturing, handling, and disposal of nanoparticles [2,46]. In many instances, since the exact mechanism of nanoparticle toxicity may not be known, it is best to work with these compounds as if they were an unknown, and with the assumption that they could be highly toxic [2]. As more information regarding the cellular toxicity of various nanoparticles becomes available, there is a substantiation of the need for more testing and greater control over the release of these nanoparticles into the environment [47]. Inorganic nanoparticles can “infect” a host, followed by transference from prey to predator moving up the food chain [47]. The source of exposure may also be important, with significant variations occurring between occupational and environmental exposures [46,48]. Currently, the argument/debate continues on how to test and regulate nanoparticle-based compounds. Some of the most rigorous legislation has taken place in Europe [48], but the United States via the Environmental Protection Agency is beginning to recognize the need for tighter controls and legislation.

Slowly, we are beginning to realize that some inorganic nanoparticles exert effects on cellular processes associated with the development of cancer. Oxidative stress, cellular apoptosis, and alterations in the p53 tumor suppressor system can all be involved either alone or together in the development of various tumors. There does not appear to be any organ system that is free from the potential toxicity of nanoparticles. Reports have shown liver toxicity [49], lung toxicity [50], reproductive system toxicity [51], and immune system toxicity [52]. Contrary to initial beliefs, over the past five years, there has been increasing evidence that inorganic oxide nanoparticles do cross the blood-brain barrier via a wide range of transport-related mechanisms [1,5,14,15,53,54,55,56,57]. Unlike conventional drugs with a molar mass of 300–350 g/mol, nanoparticles have the ability to bypass the blood-brain barrier and enter neuronal tissue via other transport systems, or via diffusion if the particle size is small enough [54]. Once the nanoparticles have entered the brain, damage follows similar mechanisms observed in peripheral organs, including oxidative stress, apoptosis, and stimulation of neuroinflammatory pathways [12,56]. Accumulation of metals in the brain is uniform across brain regions (except the olfactory bulb), and is independent of exposure time [55]. Some reported organ effects of nanoparticles directly promote tumor formation. Zinc oxide has been shown to reduce the function of the p53 tumor suppression gene, possibly via an oxidative stress mechanism, resulting in elevated tumor cell growth [58]. Direct damage to DNA may also increase the risk for tumor development if proper systems are not functioning to repair DNA damage [11]. Silver and iron oxide particles have been shown to increase the fragmentation of DNA in rats [13]. Cerium oxide can induce the generation of reactive oxygen species, resulting in oxidative damage, DNA fragmentation, and cellular apoptosis [50]. Alterations in the reproductive system may result in long-term genetic damage that would be handed down from the parents to the offspring [51].

Collectively, it has become increasingly clear in the last 5–10 years that the field of nanomaterial development and utilization is exploding and that the necessary toxicology testing and regulatory guidance is lacking. Although attempts are made to bridge the knowledge gap between function and toxicity, it is clear is that we cannot use the conventional mechanisms for assessing toxicity. Newer methods need to be developed and employed to stay ahead of the expanding curve of nanomaterial usage. Meanwhile, it is obvious that the use of nanoparticles can be very advantageous in treating certain diseases, but caution must be used when establishing the risk assessment of these materials as it relates to human exposure.

### 2.3. Metalloestrogens and Breast Cancer

Metalloestrogens (Table 2) are a class of metals shown to exert estrogenic effects through alterations of gene expression and/or modifying the activity of estrogenic receptors. There have been numerous reviews on the subject of heavy metals as potential endocrine disruptors or metalloestrogens [21,59,60,61]. With continued industrialization and a lack of mechanistic understanding regarding the use and disposal of metalloestrogens, exposure has increased steadily since the mid-20th century. Uncertainty concerning the environmental fate of metalloestrogens and an increased release of these metals has resulted in steadily elevating metalloestrogen levels in both soil and water. Once in the ecosystem, the metals freely move from consumable crops to higher biological organisms such as humans. In addition to general environmental exposure, tobacco products have been a major contributing factor to metal exposure in humans with arsenic, cadmium, chromium, copper, cobalt, lead, and mercury being reported to be in smoke condensate [62]. Previous studies have demonstrated that metalloestrogens can elicit estrogenic effects in the absence of estrogen [60,61]. This effect was surprising since the original belief was that metalloestrogens functioned as modulators of the estrogen system, requiring the presence of estrogen. Increasing evidence suggests that metalloestrogens such as cadmium can directly stimulate estrogen receptors in the absence of estrogen, leading to potentially negative health effects in postmenopausal women [22]. Further compounding health hazards are metalloestrogens’ long biological half-life (cadmium's is 10 to 30 years) and the ability to bioaccumulate [22,25]. Metalloestrogens mimic the physiologic function of estrogen and have shown an affinity for estrogen receptors. Because they can mimic estrogen, thus activating the receptor, they are considered harmful and potentially linked to breast cancer [62]. Heavy metals including copper, cobalt, nickel, lead, mercury, tin, chromium, and vanadate exhibited estrogenic activity when subjected to a variety of tests, resulting in a two- to five-fold increase in human breast cancer cell numbers [63]. In another study, two different screening systems were used to test for “estrogenicity” in heavy metals, ultimately verifying the estrogen-like activity of these metals [62,64].

**Table 2:** Examples of select metals with known or suspected estrogenic activity [22,59,60,61].

Estrogenic class	Representative metals [65]		Potential metals
**Metalloestrogens**	**Aluminum**	Copper	**Silver**
	Antimony	Lead	**Zinc**
	Arsenite	Mercury	**Titanium**
	Barium	Nickel	(some evidence—needs more study)
	**Cadmium**	Selenite	
	Chromium (II)	Tin	
	Cobalt	Vanadate	

Bold underlined: Metals are discussed in this review.

Of the “representative metals” listed in Table 2, cadmium has been shown to be estrogenic and is equipotent to the effects elicited by estradiol [26,64,66]. The majority of assays examining the effects of metalloestrogens on estrogen receptors have been performed *in vitro* using estradiol as the benchmark for estrogenic potency. The results from these studies have yielded somewhat variable data concerning potency, with effects ranging from 25%–100% of the corresponding estradiol response [27,64]. Acute *in vivo* administration of metalloestrogens (including cadmium) demonstrated the metals elicited classic estrogenic responses with increased (a) uterine weight, (b) hyperplasia, (c) hypertrophy of the endometrial lining, (d) expression and density of progesterone receptors, and (e) mammary tissue density [27,64]. A recent study that focused solely on mammary tissue development demonstrated that cadmium altered normal tissue development and gene expression with relatively high potency [63]. The authors suggested that early exposure to agents such as cadmium results in a predisposition toward breast cancer development later in life [63]. Cadmium has been shown to mimic the actions of estrogen through activation of both ERα and ERβ receptors, as well as GPR30 receptors [26]. Via the GPR30 receptor, cadmium stimulation activates MAPK, raf-1, and ERK1/2 and is a potent activator/modulator of the mitogenic effects associated with estrogen receptor stimulation [26]. Following cadmium exposure, the expression of estrogen receptors is greatly reduced [67], again mimicking the effects of natural estrogens. Cadmium can also alter estrogenic activity in tumor cells, such as the MCF-7 breast cancer cell line, leading to an increased proliferation of these tumor cell lines [68,69]. The antiestrogen melatonin has been reported to reverse the effects seen following exposure to cadmium chloride by modifying the expression and activity of hTERT, MT-1, and MT-2 in breast cancer cells [68,69]. However, results in other experimental systems are mixed, suggesting the need for more work to better to understand the estrogenic effects [70,71]. Using cadmium as the *de facto* benchmark for metalloestrogens is an excellent starting point for the toxicological investigation into the effects of other metals believed to be metalloestrogens, as well as metals which may have more indirect metalloestrogen-like effects.

## 3. Toxicity of Key Environmental Metals

### 3.1. Cadmium

Many physiological effects elicited by cadmium resemble those attributed to estrogen, which has led to cadmium being considered a “metalloestrogen” [22]. Subsequently, cadmium is the most studied of the metalloestrogens. Exposure occurs through inhalation of air containing particulate cadmium (ambient, occupational, and cigarette smoke). Normally, environmental cadmium exposure is relatively low. Higher exposure rates can occur in occupational settings (manufacturing/processing), yet exposure limits to cadmium have been lowered 50- to 100-fold in the last few decades as we increased our understanding of cadmium toxicity. A major source of cadmium exposure to humans and the contamination of soil or water is through the use of phosphate fertilizers, followed by fossil fuel combustion (automobile exhaust), iron and steel production, natural sources, cement production, cadmium-containing products, and, finally, incineration. When combined, phosphate fertilizers plus auto emissions account for over 50% of all cadmium exposure. Considering the forms of exposure and the locations of exposure to cadmium, higher concentrations of cadmium are found in males compared to females. Biochemical studies have shown that cadmium can affect steroid biosynthesis in both males and females with relatively high affinity. Alteration in steroid biosynthesis is accomplished through interference with the biosynthesis of androgens, estrogens, and progesterone [61]. The majority of published work has focused on estrogen effects compared to androgen effects. Dyer [61] and Byrne *et al.* [22] reviewed the published androgenic effects of heavy metals on testicular function. They concluded cadmium potently inhibits testosterone/5-dihydrotestosterone synthesis and function. The potency of cadmium in eliciting estrogenic effects was second only to the actions of *n*-butyltin [66]. *In vivo* studies that utilized concentrations of cadmium considered “environmentally relevant” elicited numerous changes in female rats [67]. The use of “relevant” concentrations is an issue that has plagued toxicological studies for decades. What is the toxic relevancy at a concentration 1000-fold higher than concentrations found in the environment? In females, organs/tissues that appeared to be the most sensitive to the effects of cadmium were estrogen-dependent tissues such as the mammary glands. In general, there was an observed upregulation in the development of the glands, increased milk production, and increased receptor density (estrogen and progesterone) [63]. Collectively, these changes lead to the overdevelopment of breast tissues, and potentially the development of breast cancers. Additional information regarding cadmium toxicity is discussed later since cadmium is often considered one of the prototype metals in the metalloestrogen class, and has been most strongly implicated in the development of breast cancer.

### 3.2. Aluminum

Multiple consumer products reportedly contain aluminum oxide nanoparticles [15]. Recently, there has been an increased focus on the role aluminum may have in the development of breast cancer [72,73]. Functionally, aluminum has been found to interact with DNA by binding strongly to the phosphate backbone under neutral conditions [74], suggesting that aluminum could serve as a possible source of DNA damage, thus increasing the chances of DNA mistakes and promoting the growth of the damaged cells. Aluminum can interfere with cell growth regulatory processes through many pathways, including altering gene expression [21]. An alteration in growth regulation would result in tumor development. A major question needing to be answered is whether or not aluminum plays a role in the development of breast cancer. We are exposed to aluminum on a daily basis whether it is through antiperspirants, antacids, foods, or occupational exposure. Exley *et al.* [75] demonstrated that you can measure aluminum from breast biopsy tissue and that aluminum concentration varies by region with the highest aluminum content in the outer regions of the breast. It is possible to measure aluminum concentrations in breast tissue through less invasive means, such as milk/blood [73] and nipple aspirate fluids [73,76]. The toxicity of aluminum is dependent on the breast tissue microenvironment. Focus on the microenvironment may rule out using milk or blood values unless a reliable correlation is made between milk/blood concentrations and tumor development. A method for reproducible analysis of aluminum content in breast tissue has been reported [77], yet this method uses whole breast tissue. Regardless of the measurement, the microenvironment of aluminum action appears to be very important. Locally, aluminum will interfere with iron binding to transferrin, increase oxidative stress, increase the release and actions of pro-inflammatory cytokines, and alter the ability of breast tumor cells to migrate [73,76]. The lethality of breast cancer can be due to its ability to metastasize and invade other tissue. The change in invasiveness is seen using MCF-7 breast tumor cells [78]. Since our daily aluminum burden is relatively low, a major source of aluminum exposure occurs through the use of aluminum-containing antiperspirants [65]. Although there are still many questions regarding the measurement and overall toxicity of aluminum, if the potential exists for an aluminum-containing antiperspirant/breast cancer link, then the content of aluminum in antiperspirants will need to be regulated and either reduced or eliminated [79].

### 3.3. Silver

The use of silver in medicine holds a unique position. Silver has been used in ophthalmological preparations for decades and is added as an antibacterial agent for improving the shelf-life of other drugs [1,5,14,15,80]. Little toxicological work has been done to better understand the overall toxicity of silver. As with most metals in this group, toxicity information is scarce, but the toxicity of silver nanoparticles appears to be dependent on the usual physicochemical properties of size, shape, solubility, *etc.* Proposed mechanisms of silver nanoparticle toxicity include: (a) reactive oxygen species generation; (b) independent silver ion toxicity after entering a solution; (c) endocrine disruption; and (d) inflammation and generation of pro-inflammatory cytokines [80].

Not only are silver nanoparticles in many consumer products, but the use of these preparations has spread to farming, with silver nanoparticles added for their bactericide and insecticide properties. Agricultural use has led to speculation that silver nanoparticles can make their way into the food chain and be passed on to humans. Unlike conventional pesticides that are washed off the produce, silver nanoparticles have been shown to contaminate the produce directly, and contamination can be detected inside the produce [81]. Biologically, silver nanoparticles can elicit multiple adverse effects. At concentrations that are below the level needed for the generation of reactive oxygen species, intracellular effects can still be measured. Silver nanoparticles are transported into the cell via an energy-dependent active transport system, and they do not appear to be sequestered [82]. At concentrations that would not be considered lethal to the cells, Cooper and Spitzer observed disruption of normal cytoskeleton function altering normal actin dynamics and neurogenesis [82]. At higher concentrations, size is a significant component in determining toxicity [83,84]. Exposure to smaller silver nanoparticles resulted in a greater increase in reactive oxygen species generation, and silver nitrate had virtually no effect, suggesting that the silver ion itself was not responsible for oxidative stress [84]. This work extended earlier work that reported that 20 nm silver nanoparticles were uniformly more toxic than nanoparticles at 80 and 113 nm [83]. This size-related finding in oxidative stress studies correlated to changes in pro-inflammatory marker measurements and cellular membrane integrity [83]. What is unique about the Park *et al.* [83] studies was that they compared two cell lines and reported differential sensitivity to 20 nm silver nanoparticles between fibroblasts and macrophages. Previous reports demonstrated the potential for genotoxicity and immune toxicity following exposure to silver nanoparticles [83,85,86]. Similar to other reports the smaller the size of the silver nanoparticles, the greater the toxicity that is observed. Multiple markers for genotoxicity have been examined, with consistent results being reported across all markers. Mutations in the *lacZ* gene [83], reticulocyte micronuclei, and the COMET assay in leukocytes from bone marrow [85] have all been consistent in reports that, once internalized, silver nanoparticles can damage the DNA of the host cell. Recently, these findings have been extended to the immune system and the immunotoxicity of silver nanoparticles once transported into circulating immune cells [86]. Very little work has been performed on silver nanoparticle-induced toxicity to immune cells, suggesting that due to the increasing frequency of silver nanoparticle exposure in consumer products, the need to better understand the actions of these nanoparticles on the immune system is tantamount. The toxic properties of silver can be utilized for therapeutic advantage; there are many examples of therapeutic uses for silver and silver nanoparticles. Also, its more established use is as an antibacterial agent, but recent reports have suggested that silver-containing compounds may attenuate tumor growth [87]. In polymer form, not as a nanoparticle, the silver-containing polymers were more effective at attenuating MCF-7 cell proliferation than tamoxifen [88]. Further work on silver nanoparticles and their potential as antitumor agents is promising.

An exciting prospect is that many groups are looking at “green chemistry” for the synthesis of nanoparticles ([89] for an overview). As mentioned earlier, a major concern with the production and distribution of nanoparticles is the possibility of environmental contamination. By using green chemistry to synthesize silver nanoparticles, the toxicity risk to the environment is significantly reduced and the cost of synthesis may be reduced by eliminating multiple costly steps in the synthesis process. Recently, green synthesis of silver nanoparticles was described as a biocompatible material that was used therapeutically as an anticancer therapy [90,91]. It seems that the majority of the work to date has focused on the usefulness of silver nanoparticles as anticancer agents [87,90,91,92,93,94,95,96]. The means for generating eco-friendly silver nanoparticles has been quite varied. Laboratories have been able to extract functional silver nanoparticles from unripe fruit [96], the leafy part of medicinal plants [82,93,95], strains of fungi [92] and bacteria [94]. None of these studies have taken their product to a clinical trial, but they have examined the effects of their derived silver nanoparticles on MCF-7 and MDA-MB-231 breast cancer cell lines. The evidence from these studies has suggested that green-chemistry-derived silver nanoparticles do exert a significant effect on the proliferation and viability of these cell lines. The major mechanisms for cell death appear to be through (1) reactive oxygen species generation, (2) increased activity of caspase enzymes (increased apoptosis), and (3) increased DNA fragmentation. Collectively, silver nanoparticles have demonstrated a great “therapeutic” advantage compared to other nanoparticles. The concern for potential silver nanoparticle toxicity will remain until additional work is done leading to a better understanding of the mechanism of action and toxicity associated with silver nanoparticle exposure.

### 3.4. Titanium

The major chemical form of titanium nanoparticles is titanium dioxide and it is found in many consumer products [15]. Depending on particle type, titanium may be found in sunscreens, cosmetics, and paints and coatings. Titanium nanoparticles are also being investigated for removing contaminants from drinking water [97]. Due to its general durability and lack of reactivity in a biological system, titanium is also found in many forms of implants, such as dental work, artificial joints, and as a coating on the polypropylene mesh used in breast reconstruction surgery [98,99].

The majority of existing work on titanium nanoparticles has focused on the route of entry into the body. Surprisingly, absorption from the intestinal tract is minimal and is similar to observations with larger titanium particles [100]. Inhalation of titanium nanoparticle dust could be quite hazardous. In a murine model, direct intratracheal administration produced long-term inflammation in the respiratory system by increasing Th1- and Th2-type cytokines. Collectively, there was an overall increase in the production of pro-inflammatory cytokines, recruitment of immune cells such as B-cells, and IgE production [101]. In rats, exposure to titanium nanoparticles of various sizes resulted in a size-dependent (small > large) increase in the production of pro-inflammatory cytokines, neutrophil recruitment, and cellular cytotoxicity [102]. In THP-1 cells, a more precise mechanism has been described, which includes elevation of IL-1β production via caspase-1 activation. These elevations are mediated by an increase in reactive oxygen species generation and the increased formation of cathepsin B [103].

Titanium nanoparticle damage of the respiratory tract has been well elucidated [100,101,102]. In the last decade, studies have examined titanium nanoparticle toxicity in multiple organ systems. In cells derived from areas of the mouth that would be in contact with titanium implants, titanium nanoparticle exposure resulted in increased toxicity that was size-dependent [99]. Cardiac cells exposed to titanium nanoparticles showed an increase in reactive oxygen species generation and an increased level of troponin-I and creatine kinase MB which is indicative of damage to the myocytes [104]. Central nervous system exposure to titanium nanoparticles is toxic to both neurons and astrocytes/glia. In cell cultures of immortalized neuronal (SH-SY5Y) and astrocyte (D384) cells, the results clearly demonstrated that exposure to titanium nanoparticles was toxic to both SH-SY5Y and D384 cell lines [105]. The neuronal cell line was slightly more sensitive than the astrocyte cell line but in all experimental groups, the titanium nanoparticle was more toxic than the titanium oxide bulk formulation [105]. To determine whether titanium nanoparticles can cross the blood-brain barrier following intranasal administration, Zhang *et al.* [106] measured the levels and location of the particles in various brain regions after administration. They found that particle size was a major determining factor of toxicity. Titanium nanoparticles tend to be lipophilic and displayed a differential distribution with significant levels of titanium nanoparticles detected in the cerebral cortex and the striatum [106]. Neurochemical alterations were observed with both neurotransmitters and their respective metabolites significantly affected (serotonin, dopamine, and norepinephrine), reflecting changes in neuronal activity and cellular damage that correlated with the regional distribution of the neurotransmitters [106]. Collectively, titanium nanoparticle toxicity tends to follow (a) dependence on particle size; (b) increased reactive oxygen species burden; (c) disruption of mitochondrial/cellular membrane; (d) increased recruitment of pro-inflammatory cells and cytokines; and (e) genotoxicity indicated by DNA fragmentation, micronuclei assay, chromatid exchange, *etc.* [107,108].

Similar to the action of silver nanoparticles, titanium nanoparticles may also have a positive therapeutic action in addition to the observed toxicity. A great deal of interest exists in the ability of titanium compounds to halt tumor growth [109]. Titanium exists in many chemical forms, and in addition to titanium oxide associated with nanoparticles, there are also other complexes containing titanium such as *bis*(β-diketonato)titanium complex, and multiple titanocene complexes. Each compound has its unique chemico-biological characteristics, most of which do not follow conventional models [109]. Complexes within the same chemical class may have opposing actions. For example, one form of titanocene exhibits a strong proliferative effect on MCF-7 cells whereas another titanocene complex is strongly cytotoxic under the same assay conditions [110]. Using the “anti-proliferative” titanocene complexes as a model, newer formulations of titanocene complexes have shown to have strong antiproliferative actions and to be strongly cytotoxic to both MCF-7 and SkBr3 breast cancer cell lines [111]. Unlike other metals that utilize the oxide form as the nanoparticle, the use of titanium has provided a series of very unique and innovative ways to combat cancer growth and metastasis. It is understood that titanium may generate reactive oxygen species which are toxic to tumor cells [112]. Research advancements have progressed to examining the oxidative-reductive microenvironment of the tumor, followed by a smart design of the titanium complex utilizing the microenvironment to destroy the tumor cells [113]. Other forms of titanocene have been developed to function similar to a time-release drug. The titanocene complex forms a hybrid nanofibrous scaffold that is slowly released over time in a tightly controlled and localized area [114]. By binding titanium oxide particles to avidin proteins, the proteins preferentially bind to cancer cells. The titanium-avidin complex provides a targeted area with a localized higher concentration of titanium oxide nanoparticles. Administration of external ultrasound radiation promotes the generation of reactive oxygen species from the titanium nanoparticles, damaging or destroying the tumor cells in a very precise fashion [115]. Similar to ultrasound radiation, titanium nanoparticle transport concentrates titanium within the cell, and when the titanium particles are photocatalyzed, they result in significant cellular damage and death. Photocatalyzed cell death is observed via activation of the apoptosis pathways in highly malignant MDA-MB-468 breast cancer cells, whereas the less-malignant MCF-7 cells were unaffected [116]. These findings suggest that development of customized titanium nanoparticles for specific tumors is possible. Specialized titanium nanoparticles are similar to the current trend in specialized medicine that is more frequently utilized in cancer patients.

### 3.5. Silica or Silicon Oxide

Occasionally, the terms “silica” and “silicon” are used interchangeably, which is incorrect. Silica is derived from silicon and is “silicon oxide”. A significant number of preparations are currently available which contain silica (or silicon oxide). Interestingly, silica appears to have multiple uses regarding the treatment of breast cancer [117,118,119], yet in a slightly different chemical form it can be highly toxic [120,121,122,123].

Silica toxicity is dependent on the form/function of the silica nanoparticle [121,122]. In its simplest chemical form, silica exposure has been shown to increase the (1) production of reactive oxygen species, (2) DNA damage, and (3) inflammation [123]. In the basic chemical state, silica exposure has also been shown to result in liver toxicity [120]. Functionalizing the silica by conversion to a mesoporous or amorphous silica reverses the damaging effects observed after exposure [121,123]. It is this conversion of silica nanoparticles to amorphous/mesoporous that not only reduces the damaging effects following silica exposure but permits the use of silica as a therapeutic agent. Using mesoporous silica nanoparticles, drugs or other therapeutic agents are combined with silica [117,118,124]. In this manner, the silica-drug combination acts like a “Trojan horse” and is readily taken into the tumor cells [118,124,125]. The potential also exists for using mesoporous silica to overcome tumor drug resistance [117]. Similar to observations with silver and titanium nanoparticles, the modification of the chemical structure of silica can “flip” its biological action from toxic to therapeutic.

In additional to potential use as a therapeutic agent (or as an adjuvant therapeutic agent), engineered silica nanoparticles have demonstrated utility in the detection and identification of breast cancer cells. Using a fluorescent dye and antibodies against Human Epidermal Growth Factor Receptor 2 (HER2) conjugated to silica nanoparticles, it is possible to isolate and label high-affinity breast cancer cells that are positive for HER2/neu [126]. Even with this early test, it was considered to be highly sensitive with a limit of detection of HER2/neu of 1 ng/mL and a large linear range of detection of 1 ng/mL–10 µg/mL [126]. A recent adaptation by Jo *et al.* [119] uses aptamer-functionalized silica nanoparticles, which will identify HER2/neu and/or mucin 1. This platform allows for specialized identification of cancer cells and is even more sensitive than earlier detection methods with a lower limit of detection estimated to be 1 cell/100 µL. It is clear that under the appropriate conditions, silica use in breast cancer will be more advantageous than detrimental. Engineered silica has demonstrated characteristics that may result in its use as a potential cancer therapy. Non-engineered silica exposure still exhibits cellular toxicity through multiple mechanisms and care must be taken to minimize exposure.

### 3.6. Zinc

Zinc is one of the widely used metals for nanoparticle production, yet is one of the least toxicologically studied. Zinc in its oxide form (ZnO) is found in many consumer items, from cosmetics to paint, to sunscreens. Toxicity studies on zinc oxide have been scarce, and the studies that do exist are in invertebrates, or have yielded mixed/conflicting results. For example, there is some question as to whether or not the zinc ion itself is responsible for toxicity [127] or if the zinc must be in the nanoparticle form [128]. Whether the concentration or the size of the zinc nanoparticle is most important for eliciting toxicity is unclear, but that is in conflict with the existing dogma regarding nanoparticle toxicity being size-dependent [128]. The pathway leading to toxicity involves reactive oxygen species, apoptosis, and inflammation regardless of the model system studied [58,83,129]. In an aquatic environment, zinc nanoparticle exposure resulted in the development of abnormalities in frogs [130]. The level of toxicity was comparable to that observed following copper exposure with a lower threshold of approximately 3 ppm (3 mg/L) [130]. In a bee model, the zinc oxide nanoparticle displayed no adverse effects, whereas the presence of zinc ions elicited developmental deficits [127].

What does appear consistent is that reactive oxygen species are important for zinc-related toxicity. Whether it is the size of the particle or the concentration, an increase in reactive oxygen species generation is observed [58,83,129]. Following reactive oxygen species generation, increased production and release of pro-inflammatory cytokines (IFN-γ, TNF-α, and IL-12) has been described [129], as well as the increased activity of the p53 apoptosis pathway [58]. Collectively, the end-point of these cellular responses is damage/death in the exposed cell(s). It is clear that with zinc nanoparticles, additional work is needed for a better understanding of the toxicity (or lack of toxicity) associated with exposure to zinc nanoparticles.

## 4. Direct Mechanisms for Metal Involvement in Breast Cancer

Normal physiological functioning requires the proper expression and activity of estrogen receptors. It is clear that estrogen receptors play essential roles in the normal growth and differentiation of breast tissue as well as the prognosis of human breast cancer. As toxicological studies improve, and more metals are studied for endocrine-related adverse effects, it is apparent that many metals can elicit profound physiological effects within a living organism. A high level of significance has been placed on whether or not metals fall into the metalloestrogen class. Metalloestrogen metals will elicit estrogenic effects and potentially change the breast anatomy. Alterations in breast anatomy correlate with increased susceptibility to the development of cancer. Changes in anatomy include early onset of puberty, an increase in epithelial area, and an increase in the number of terminal end buds in the mammary gland [24]. The correlation between breast cancer and heavy metals has expanded to include numerous metals that were identified in breast biopsies in significant concentrations. Recent studies have drawn a significant correlation between specific metals and their presence in cancerous breast tissues, which is not observed when compared to biopsies from healthy breast tissue [131,132]. In other studies, the ability of cadmium or other metals to directly correlate with breast cancer falls short, contributing to confusion that many of these metalloestrogens have regarding the development of breast cancer [133]. The majority of data from *in vitro* assays has been convincing, suggesting that cadmium can function as a very potent metalloestrogen. The actions of cadmium have provided the most compelling evidence of a correlation with breast cancer proliferation [25]. Comparing the affinities of estrogen and cadmium for the estrogen receptor (ERα), both compounds exhibit similar affinities (0.4–0.5 nM), yet defining cadmium action at the ERα binding using *in vitro* ligand binding assays has yet to be determined [25]. The prevailing hypothesis is that ERα is sequestered in the inactive form—low estrogen or no estrogen. Once activated, the receptor undergoes a conformational change and enters the active state. Increasing the number of active ERα receptors will increase estrogen responsiveness [22]. Cadmium inhibits the action of estrogen to increase the number of active ERα suggesting that cadmium interferes with estrogen binding to its recognition site on the ERα receptor. Molecular studies further support this hypothesis by examining the effects of point mutations and selective estrogen ERα antagonists to block estrogen effects in transfected COS-7 [134]. Site-directed mutagenesis has identified possible recognition sites for these effects, which include cys381, cys447, glu523, his524, and asp538 [22,134]. Multiple investigators have demonstrated the direct action of cadmium on estrogen receptors, plus the activation of various protein kinases, and the increased proliferation of breast cancer cells. In humans, these findings are not quite as clear. Studies have suggested a correlation between cadmium content and breast cancer [131,132], yet Silva *et al.* [133] reviewed numerous studies where the data are inconclusive. Although the data are not definitive for being a causative agent, cadmium can be considered a risk factor for breast cancer development. Extrapolating the findings from *in vitro* assays to physiological effects in humans has been difficult and sometimes elusive. *In vitro* assays commonly use relatively high concentrations of the metal and the exposure time can be considered “acute” compared to human exposure. In humans, a metal such as cadmium enters the body at low concentrations but can sequester in various tissues and maintain a presence for years. This low-level, constant, chronic exposure would be a more accurate model. Potential bioaccumulation and increased metal burden over time, as well as the potential presence of many other estrogenic toxins, have been difficult to account for in designed laboratory environments. Real-world exposure would involve a very gradual change in intracellular signaling systems, leading to changes that may lead to indirect changes that are not being observed in the current *in vitro* experimental paradigm.

## 5. Discussion and Conclusions

It is apparent that the field of metalloestrogens and nanotoxicology will be undergoing a rapid advancement in the next few years. There have been calls to increase the funding for additional toxicology testing of endocrine-disrupting chemicals, such as the metalloestrogens [135]. The area of nanotoxicology is underdeveloped and an increasing number of investigators have been assessing the toxicological properties of nanoparticles [136]. The information that has come from these studies has been confusing, concerning the question “What makes a nanoparticle toxic?” Use of the growing field of bioinformatics has been suggested to expedite the toxicity testing of many nanoparticles [137]. The use of known physicochemical information regarding a nanoparticle, predictions can be made which will assist in determining the particles' toxicity. However, as discussed earlier, there is a significant variation between different nanoparticles and whether it is their size, shape, or concentration that is the major factor responsible for their toxicity. Mechanisms of nanoparticle toxicity must be elucidated, there are many nanoparticles in consumer products that are commonly used, and there is an increasing use of these particles as therapeutic agents.

What we know or have an understanding of is:

- The toxicity of metalloestrogens is real, and there are metals (such as cadmium and aluminum) that are potent metalloestrogens, and are also used in some formations of nanoparticles. One concern is whether it is the ion or the nanoparticle that exerts the biological effect. Although some evidence suggests nanoparticles can be toxic, there is more data available describing the toxic effects of various metal ions, leading to extrapolating toxic effects from metal ions to nanoparticles. Therefore, it may be a combination of cellular effects that is observed.
- There is data for other metals (such as silver and titanium) that is equivocal at best regarding the metals' effects on estrogen receptors. Initially, the focus was the actions of metal ions at estrogen receptors, and their ability to alter estrogenic effects on breast tissue. It is now increasingly clear that many metals exert cellular effects through multiple systems, and the combination of effects will alter cellular function.
- There is a possibility that some metals/nanoparticles exert indirect effects through cellular signaling systems to promote the formation of tumors. Many of the metals examined have been shown to increase production of reactive oxygen species and DNA fragmentation. Both responses may lead to aberrant cell growth and cancer development.
- Not all metals used in nanoparticle formation cause cancer; in fact, some metals have demonstrated usefulness in combating cancer by being actively taken into cancerous cells where they damage the intracellular machinery of the cell, resulting in cellular death [138].

Overall, this is a highly dynamic and growing area for the foreseeable future. There is a significant need to establish metalloestrogen toxicity and their association with the development of nanoparticles as potential therapeutic agents.
